# Isolation, Characterization and Draft Genome Analysis of Bacteriophages Infecting *Acidovorax citrulli*

**DOI:** 10.3389/fmicb.2021.803789

**Published:** 2022-02-03

**Authors:** Katarina Gašić, Mina Obradović, Nemanja Kuzmanović, Nevena Zlatković, Milan Ivanović, Danijela Ristić, Aleksa Obradović

**Affiliations:** ^1^Institute for Plant Protection and Environment, Belgrade, Serbia; ^2^Institute of Molecular Genetics and Genetic Engineering, University of Belgrade, Belgrade, Serbia; ^3^Federal Research Centre for Cultivated Plants, Institute for Plant Protection in Horticulture and Forests, Julius Kühn-Institut, Braunschweig, Germany; ^4^Faculty of Agriculture, University of Belgrade, Belgrade, Serbia

**Keywords:** *Acidovorax citrulli*, bacteriophage, genome analysis, host specificity, phage therapy, biocontrol

## Abstract

Bacterial fruit blotch and seedling blight, caused by *Acidovorax citrulli*, is one of the most destructive diseases of melon and watermelon in many countries. Pathogen-free seed and cultural practices are major pillars of the disease control. However, use of bacteriophages as natural biocontrol agents might also contribute to the disease management. Therefore, we isolated 12 bacteriophages specific to *A. citrulli*, from phyllosphere and rhizosphere of diseased watermelon plants. The phage strains were characterized based on their host range, plaque and virion morphology, thermal inactivation point, adsorption rate, one step growth curve, restriction fragment length polymorphism (RFLP), and genomic analysis. Transmission electron microscopy of three phage strains indicated that they belong to the order *Caudovirales*, family *Siphoviridae.* All phages lysed 30 out of 32 tested *A. citrulli* strains isolated in Serbia, and did not lyse other less related bacterial species. They produced clear plaques, 2 mm in diameter, on bacterial lawns of different *A. citrulli* strains after 24 h of incubation. The thermal inactivation point was 66 or 67°C. They were stable at pH 5–9, but were sensitive to chloroform and inactivated in either 5 or 10 min exposure to ultraviolet (UV) light. RFLP analysis using *Eco*RI, *Bsm*I and *Bam*HI enzymes did not show genetic differences among the tested phages. Adsorption rate and one step growth curve were determined for the *Acidovorax* phage ACF1. Draft genome sequence of the ACF1 phage was 59.377 bp in size, with guanine-cytosine (GC) content 64.5%, including 89 open reading frames. This phage shared a very high genomic identity with *Acidovorax* phage ACPWH, isolated in South Korea. Evaluation of systemic nature of ACF1 strain showed that it can be absorbed by roots and translocated to upper parts of watermelon plants where it survived up to 10 days.

## Introduction

Bacterial fruit blotch (BFB) of cucurbits is a disease caused by *Acidovorax citrulli*, a bacterium which can lead to serious yield and seed production losses in cucurbit crops. In several occasions, when conditions favored bacterial dissemination in the United States, watermelon crop damage reached 100% (Latin and Rane, 1990). Typical symptoms of BFB in watermelon, such as seedling water-soaked lesions and blotches on fruits, have been noticed since the mid-1960s (Webb and Goth, 1965) but the pathogen responsible for the disease was first identified by Latin and Hopkins (1995). During the 1990s, BFB rapidly progressed toward other crops such as honeydew, pumpkin, and cucumber (Isakeit et al., 1997; Langston et al., 1999; Martin et al., 1999).

*Acidovorax citrulli* is a Gram-negative bacterium primarily disseminated by infected cucurbit seed (Rane and Latin, 1992). Usually, first symptoms appear on seedlings and young plants. The symptom development and spread of the infection are facilitated by the transplant growth conditions in nurseries. It has been shown that, under such conditions, a single seed containing 10 *A.citrulli* colony forming units (CFU) within a seed lot can lead to BFB transmission (Dutta et al., 2012a). The bacterium resides on the surface and underneath the seed coat, and if the seed infection originates from the blossom invasion, *A.citrulli* cells are deposited deep within the seed, which hinders their control (Dutta et al., 2012b).

*Acidovorax citrulli* has been detected in Serbia for the first time on watermelon plants in 2014 (Popović and Ivanović, 2014), causing significant economic loses. Although, it has been eradicated, sporadic occurrence of BFB was recorded in upcoming years, affecting watermelon production in main production regions in Serbia (Zlatković et al., 2015, 2017).

Different management strategies for BFB control have been attempted, including selection of the disease resistant or tolerant cultivars (Bahar et al., 2009), treatments with peroxyacetic acid (Hopkins et al., 2003) and chitosan solution (Li et al., 2013), as well as use of biological control approaches such as non-pathogenic *A. citrulli* strain (Johnson et al., 2011) or antagonistic rhizobacteria (Adhikari et al., 2017). Still, BFB is a considerable threat to watermelon and melon production worldwide (Burdman and Walcott, 2012).

Bacteriophages, viruses of bacteria, are considered promising biocontrol agents (Buttimer et al., 2017; Choudhary et al., 2022). Previous studies investigated their potential in use against different genera of phytopathogenic bacteria as previously reviewed (Jones et al., 2007; Buttimer et al., 2017; Stefani et al., 2021). Recently, Rahimi-Midani et al. (2018) reported for the first time isolation of phages infecting *A. citrulli.*

The aim of the present study was to isolate bacteriophages specific to *A. citrulli*, study their characteristics and evaluate possibilities of their use in biocontrol of BFB.

## Materials and Methods

### Bacterial Strains and Culture Conditions

All bacterial strains used in this study (Table 1) were stored at −20 or −80°C in Nutrient Broth (NB) supplemented with 20 and 30% of glycerol, respectively. The strains were grown on Nutrient agar (NA) or King’s medium B (KB) 24 h prior to use. Bacterial suspensions were prepared in sterile distilled water, and concentration was adjusted to 5 × 10^8^ (OD_600_ = 0.3) and then diluted accordingly.

**TABLE 1:** The host range of studied bacteriophages.

Bacterial strain	Bacterial species	Host	Origin	Year of isolation	Acidovorax phages ACF1 –ACF12
KBI76	*Acidovorax citrulli*	*C. lanatus*	Ašanja, Serbia	2014	+
KBI77	*Acidovorax citrulli*	*C. lanatus*	Ašanja, Serbia	2014	−
KBI78	*Acidovorax citrulli*	*C. lanatus*	Ašanja, Serbia	2014	−
KBI79	*Acidovorax citrulli*	*C. lanatus*	Ašanja, Serbia	2014	+
KBI80	*Acidovorax citrulli*	*C. lanatus*	Ašanja, Serbia	2014	+
KBI81	*Acidovorax citrulli*	*C. lanatus*	Ašanja, Serbia	2014	+
KBI82	*Acidovorax citrulli*	*C. lanatus*	Ašanja, Serbia	2014	+
KBI83	*Acidovorax citrulli*	*C. lanatus*	Ašanja, Serbia	2014	+
KBI84	*Acidovorax citrulli*	*C. lanatus*	Ašanja, Serbia	2014	+
KBI85	*Acidovorax citrulli*	*C. lanatus*	Ašanja, Serbia	2014	+
KBI86	*Acidovorax citrulli*	*C. lanatus*	Ašanja, Serbia	2014	+
KFB340	*Acidovorax citrulli*	*C. lanatus*	Ašanja, Serbia	2014	+
KFB341	*Acidovorax citrulli*	*C. lanatus*	Ašanja, Serbia	2014	+
KFB342	*Acidovorax citrulli*	*C. lanatus*	Ašanja, Serbia	2014	+
KFB343	*Acidovorax citrulli*	*C. lanatus*	Ašanja, Serbia	2014	+
KFB344	*Acidovorax citrulli*	*C. lanatus*	Ašanja, Serbia	2014	+
KFB345	*Acidovorax citrulli*	*C. lanatus*	Ašanja, Serbia	2014	+
KFB346	*Acidovorax citrulli*	*C. lanatus*	Ašanja, Serbia	2014	+
KFB347	*Acidovorax citrulli*	*C. lanatus*	Ašanja, Serbia	2014	+
KFB348	*Acidovorax citrulli*	*C. lanatus*	Ašanja, Serbia	2014	+
KFB349	*Acidovorax citrulli*	*C. lanatus*	Ašanja, Serbia	2014	+
KFB350	*Acidovorax citrulli*	*C. lanatus*	Ašanja, Serbia	2014	+
KFB351	*Acidovorax citrulli*	*C. lanatus*	Čelarevo, Serbia	2014	+
KFB352	*Acidovorax citrulli*	*C. lanatus*	Čelarevo, Serbia	2014	+
KFB365	*Acidovorax citrulli*	*C. lanatus*	Rečka, Serbia	2015	+
KFB366	*Acidovorax citrulli*	*C. lanatus*	Rečka, Serbia	2015	+
KFB367	*Acidovorax citrulli*	*C. lanatus*	Rečka, Serbia	2015	+
KFB368	*Acidovorax citrulli*	*C. lanatus*	Rečka, Serbia	2015	+
KFB370	*Acidovorax citrulli*	*C. lanatus*	Šabac, Serbia	2016	+
KFB371	*Acidovorax citrulli*	*C. lanatus*	Šabac, Serbia	2016	+
KFB372	*Acidovorax citrulli*	*C. lanatus*	Šabac, Serbia	2016	+
NCPPB4156	*Ralstonia solanacearum*	*Solanum tuberosum*	Netherlands	1995	−
NCPPB 3679*^T^*	*Acidovorax citrulli*	*Citrullus lanatus*	United States	unknown	−
NCPPB 2968	*X. euvesicatoria*	*Capsicum frutescens*	United States	1977	−
NCPPB 1423	*X. vesicatoria*	*Lycopersicon esculentum*	Hungary	1957	−
NCPPB 4321	*X. gardneri*	*Lycopersicon esculentum*	Serbia	1953	−
NCPPB 881	*X. perforans*	*Lycopersicon esculentum*	United States	1991	−
KBI32	*E. amylovora*	*Cydonia oblonga*	Serbia	2013	−
KFB68	*Pectobacterium carotovorum* ssp. *carotovorum*	*Brassica oleracea* var. *capitata*	Serbia	1999	−
KBI05	*Dickeya ssp.*	*Solanum tuberosum*	United Kingdom	unknown	−
KBI018	*Clavibacter michiganensis* ssp. *michiganensis*	*Lycopersicon esculentum*	Hungary	1957	−
KBI04	*C. m.* ssp. *sepedonicus*	*Solanum tuberosum*	Finland	1983	−
C58	*Agrobacterium tumefaciens*	*Prunus cerasus*	United States	1958	−
GSPB 1142	*Pseudomonas syringae* pv. *syringae*	*Phaseolus* sp.	Germany	1967	−
A1	*Pseudomonas graminis*	Apple phyllosphere	Serbia	2015	−
A2	*P. graminis*	Apple phyllosphere	Serbia	2015	−
A3	*P. graminis*	Apple phyllosphere	Serbia	2015	−
A4	*P. graminis*	Apple phyllosphere	Serbia	2015	−
B130	*Pseudomonas fluorescens*	Ji et al., 1996	–	–	−

*+, clear plaque; − absence of plaque. KBI – Collection of bacteria, Institute for Plant Protection and Environment, Belgrade; KFB – Collection of phytopathogenic bacteria, University of Belgrade, Faculty of Agriculture, Belgrade; NCPPB – The National Collection of Plant Pathogenic Bacteria, FERA, United Kingdom.*

For phage detection and propagation, semisolid nutrient agar yeast extract medium (NYA; 0.8% NB, 0.6% agar, and 0.2% yeast extract) (Balogh et al., 2003) or NB was used. Phage concentration was determined by serial dilutions of phage suspension in sterile tap water or SM buffer (10 mM Tris–HCl, pH 7.5; 100 mM NaCl; 10 mM MgSO_4_), followed by a plaque assay on NYA medium by using *A. citrulli* strain KBI 86 as a host, as previously described (Gašić et al., 2011, 2018).

### Isolation, Purification and Propagation of Phages

Samples for phage isolation were collected from the watermelon field affected by *A. citrulli* during 2014. Watermelon plants and fruits showing symptoms of BFB as well as watermelon rhizosphere soil were used for phage isolation by enrichment method as follows: a flask containing 50 ml NB with 2.5 g CaCO_3_ was inoculated with 5 ml suspension of *A. citrulli* strain KBI 86 in sterile distilled water (conc. 10^8^ CFU/ml), followed by addition of 10 g of soil or 5 g of plant tissue; samples were incubated overnight on a horizontal rotary shaker (100 rpm) at 27°C; ten ml aliquots were centrifuged (10,000 *g*, 10 min), and resulting supernatant was filter sterilized to remove any remaining bacterial cells, and stored at 4°C.

The presence of specific phages in the suspension was tested by screening for lysis of the target bacterium *A. citrulli* strain KBI 86. Suspension of bacteria (100 μl, conc. 10^9^ CFU/ml) was pipetted in the center of the empty Petri dish, followed by adding NYA medium (cooled to 48°C). Bacteria and medium were mixed by horizontal rotation of the plates. After medium solidified, 10 μl of tested suspensions was spotted on the surface of the medium and after 48 h of incubation appearance of plaques indicating lysis of the bacterial cells within the inoculated area were scored.

Phage purification was performed by three subsequent single plaque isolation steps as previously described (Gašić et al., 2011). Concentration of phage in suspension was determined by plating 100 μl of ten-fold dilution of purified phage suspension and 100 μl of suspension of *A. citrulli*, strain KBI 86 in NYA medium.

Phage propagation was performed by inoculation of actively growing culture of *A. citrulli* strain KBI 86 (conc. 10^8^ CFU/ml) in NB, at the multiplicity of infection (MOI) of 0.1. After 24 h of incubation on the rotary shaker (150 rpm, 27°C), culture was filtered through a 0.22 μm membrane filter and stored either at 4°C, or for long term storage in NB containing 30% glycerol at −80°C (Gašić et al., 2011).

### Host Range Analysis

The ability of *A. citrulli* specific phages to lyse bacterial strains isolated from watermelon samples collected from different regions in Serbia was investigated. Twelve phages were tested against 32 *A. citrulli* strains including *A. citrulli* type strain NCPPB 3679*^T^*, as well as 22 strains of other phytopathogenic or saprophytic bacterial species. Phage suspensions (5 μl), prepared in sterile tap water, were spotted onto the surface of solidified NYA medium mixed with suspensions of selected bacterial strains. After 24 h of incubation at 27°C, plaque formation was observed. The test has been repeated three times.

### Transmission Electron Microscopy

Morphology of *Acidovorax* phages ACF1, ACF8, and ACF12 was observed by transmission electron microscopy (TEM) using the negative staining protocol with 1% uranyl acetate solution, as previously described (Balogh, 2006; Gašić et al., 2011). The phages were observed and photographed by TEM (JEOL JEM-1400 series) with an accelerating voltage of 120 kV. Phage dimensions were evaluated by measurement of head diameter and length of the tail, and calculated as an average value of five phage particles for each isolate.

### Thermal Inactivation of Phages

Thermal point of inactivation was studied for three *Acidovorax* phages, ACF1, ACF8, and ACF12. In test tubes, 1 ml of each phage suspension (10^7^ PFU/ml) in sterile tap water was exposed to temperatures ranging from 30 to 80°C, with intervals of 10°C, for 10 min in a water bath. The second repetition of the test was done in the temperature range from 61 to 70°C, with an interval of 1°C. After incubation, tubes with phage suspensions were transferred to 20°C water bath, in order to stop the extended effect of the temperature treatment. Viability of phages after different temperature treatments was assayed by the spot test on NYA medium with *A. citrulli* strain KBI 86. Plaque formation was observed after 20 h of incubation at 27°C. The temperature at which no viable phage particles capable of lysing bacterial cells were detected was defined as the thermal inactivation point.

### The Effect of Ultraviolet Irradiation on Phage Viability *in vitro*

The effect of ultraviolet (UV) irradiation on three phage strains ACF1, ACF8, and ACF12 was studied. As a source of UV light, UV lamp integrated in the laminar flow hood was used (Philips TUV G30T8, UVC = 254 nm). Suspensions of phages were prepared in sterile tap water to the concentration of 10^5^ PFU/ml and 10 ml was poured into each Petri dish. Suspensions were exposed to UV irradiation from the distance of 50 cm and for the duration of 5 and 10 min (Czajkowski et al., 2014). After the treatment, the titer of phages in the suspensions was assayed. In the second repetition of the experiment, the suspensions were exposed to the irradiation for either 2 or 5 min.

### The Effect of Chloroform on Phage Vitality

Suspensions of phages ACF1, ACF8, and ACF12 (1 ml of 10^6^ PFU/ml), were incubated on a shaker with or without the supplement of 20% chloroform for 1 h. Afterward, the phage titer in the suspensions was checked by plating decimal dilutions of the suspensions as described above. The experiment was repeated twice.

### The Effect of pH on Phage Survival

For testing the effect of different pH values on phage survival, suspensions of three phage strains ACF1, ACF8, and ACF12 (10^4^ PFU/ml) were prepared in SM buffer of different pH values (2, 5, 7, 9, and 11), and incubated for 24 h at room temperature, followed by the phage titer assay. The experiment was repeated twice.

### Optimal Multiplicity of Infection

Optimal multiplicity of infection (MOI) of phage ACF1 was determined as previously described by Gašić et al. (2011), with slight modification. In order to eliminate bacterial cells, the phage suspensions were filtered by using 0.22 μm pore size filter (Sarstedt, syringe filter R-33 mm) instead of the chloroform treatment (10% v/v), after which the titer of multiplied phages was determined.

### Adsorption Rate

Adsorption rate, i.e., a number of phage particles adsorbed to the host cell in particular time, was determined as described previously (Ellis and Delbrück, 1939; Gašić et al., 2011). Moreover, percentage of *Acidovorax* phage ACF1 particles adsorbed to the host *A. citrulli* KBI 86 was determined. The experiment was repeated three times.

### One Step Growth Curve

In order to determine parameters of phage life cycle, a modification of the protocols of Ellis and Delbrück (1939) and Carlson (2005) was used, as described by Gašić et al. (2011). Briefly, *A. citrulli*, strain KBI 86 was grown in 40 ml NB at 27°C, with shaking until it reached the concentration 10^8^ CFU/ml (OD_600_ = 0.3). One ml of the culture was transferred to a sterile microtube and 10 μl of ACF1 phage suspension (10^8^ PFU/ml) was added. The mix was incubated for 20 min at 27°C, to allow the adsorption of phages to the bacterial cell surface, and afterward was diluted 10,000 times in NB. During further 110 min of incubation, starting from the moment of dilution, 100 μl of the suspension was collected every 10 min and phage titer was determined. Latent period, rise period and burst size was calculated as previously described (Gašić et al., 2011). The experiment was repeated three times.

### Bacteriophage DNA Extraction

For genomic DNA extraction, 35 ml of phage suspension (conc. 10^9^ PFU/ml) was centrifuged (28,000 *g*, 90 min, 4°C), and pellet containing phages was resuspended in 700 μl of SM buffer. In order to eliminate any bacterial nucleic acid, samples were treated with 10 μl/sample DNase I (1 U/μl) and 1 μl/sample RNase A (100 mg/ml) at 37°C for 60 min. DNA extraction was further performed as previously described by Lehman et al. (2009). DNA quality and quantity was assessed by NanoDrop (NanoPhotometer^®^ N60), while DNA integrity was assessed by agarose (0.8% agarose) gel electrophoresis.

### Restriction Fragment Length Polymorphism Analysis

DNA of three phages (ACF1, ACF8, and ACF12) was digested with *Bam*HI, *Eco*RI, and *Bsm*I restriction enzymes (Fermentas, Lithuania) as recommended by manufacturer. Digestion reaction contained 500 ng DNA, 1.5 μl buffer, 2 μl restriction enzyme and up to 15 μl nuclease-free water. The mixtures were incubated at 37°C for 16 h. DNA fragments were separated by agarose (1%) gel electrophoresis in Tris-acetate-EDTA buffer, stained with Midori Green (MIDORI Green Advance, NIPPON Genetics EUROPE) 2% (v/v) and visualized by a digital imaging camera (Vilber Lourmat, France).

The *in silico* restriction fragment length polymorphism (RFLP) analysis was performed by pDRAW32 software (AcaClone Software)^1^.

### Phage Genome Sequencing and Bioinformatic Analysis

Genome of *Acidovorax* phage ACF1 was sequenced using the Illumina HiSeq 2500 system with paired-end reads at BaseClear, Leiden, Netherlands following the manufacturer’s instructions. Initial quality assessment was based on data passing the Illumina Chastity filtering. Subsequently, reads containing PhiX control signal were removed using an in-house filtering protocol. In addition, reads containing (partial) adapters were clipped (up to minimum read length of 50 bp). The second quality assessment was based on the remaining reads using the FastQC quality control tool version 0.10.0. The quality of the FASTQ sequences was further enhanced by trimming off low-quality bases using the “Trim sequences” option of the CLC Genomics Workbench version 9.5.1 (Qiagen, Aarhus, Denmark). The *de novo* assembly was performed using the “*De novo* assembly” option of the same software. Misassemblies and nucleotide disagreement between the Illumina data and the contig sequences are corrected with Pilon version 1.20 (Walker et al., 2014). The accuracy of the assembly was checked by mapping reads back to contigs followed by visual inspection.

The assembled genome was annotated using RAST (Aziz et al., 2008; Overbeek et al., 2014; Brettin et al., 2015), Prokka (Seemann, 2014), and DFAST^2^ (Tanizawa et al., 2018) tools. Functional bioinformatic annotation for predicted ORF gene products was refined manually using BLASTP^3^, Pfam^4^ (Finn et al., 2010), and Virfam searches^5^ (Lopes et al., 2014). The potential presence of virulence-associated genes and antibiotic resistance genes in the phage genome was analyzed by VirulenceFinder v. 2.0^6^ and ResFinder ver. 4.1^7^ (Kleinheinz et al., 2014). The presence of transfer RNA genes was assessed using ARAGORN^8^ (Laslett and Canback, 2004) and tRNAscan-SE^9^ (Lowe and Chan, 2016). The genome sequence was compared to other viruses using BLAST analysis (Altschul et al., 1990) and PASC web tool (Bao et al., 2014), while comparisons at the amino acid level were done using CoreGenes 3.5 (Zafar et al., 2002).

Phage genome comparison and its visualization was performed using Easyfig 2.2.5 (Sullivan et al., 2011). The genomic relatedness between phage genomes was performed by calculating average nucleotide identity (ANI) values. For this purpose, we used the JSpecies Web Service and employed blast algorithm (Richter et al., 2016). Phylogenetic analysis was carried out based on the amino acid sequences of the gene encoding DNA polymerase of the *Acidovorax* phage ACF1 and 24 members of *Siphoviridae* viruses found in the GenBank database, using a BLASTP *E*-value cutoff of 1e-03. The sequences were aligned using MAFFT v7 (Katoh and Standley, 2013). A maximum-likelihood phylogenetic tree was constructed using IQ-TREE v1.6.12 (Nguyen et al., 2015) with 1000 ultrafast bootstrap replications (Hoang et al., 2017) and LG + I + G4 substitution model suggested by ModelFinder (Kalyaanamoorthy et al., 2017).

### Nucleotide Sequence Accession Number

The genome sequence of the *Acidovorax* phage ACF1 has been deposited at DDBJ/ENA/GenBank under the accession MZ547449 and BioProject PRJNA745195. The raw sequencing reads were deposited in the Sequence Read Archive (SRA) under the same BioProject PRJNA745195.

### Phage Translocation in Watermelon Plants

The possibility of phages to be absorbed through the root system of plants and their further translocation through the plant vascular system has been studied based on experiments by Iriarte et al. (2012). The experiment was conducted three times. Commercial watermelon seeds cv. Crimson sweet (Hoya Seed) were planted and cultivated in a plant growth chamber, at 27°C day (16 h) and 15°C night (8 h) temperature. Two weeks after sprouting, the plants were drenched with 100 ml suspension of phage ACF1 at concentration 1.6 × 10^8^ PFU/ml (first test) or 75 ml of phage suspension concentration 1.4 × 10^9^ PFU/ml (second and third test). Total number of treated plants per assay was 21. Control group was treated with tap water in the same manner. Plants were sampled seven times: after 1, 2, 3, 5, 7, 10, and 14 days from the treatment. In each sampling, three treated and three control plants were dissected. The plants were carefully uprooted and divided into three sections: root, hypocotyl with cotyledons and foliage. Tissue of each section was homogenized by mortar and pestle in sterile distilled water (1 ml per 1 g of tissue) and filtered through a syringe filter (Sarstedt, R - 33 mm, pore size 0.22 μm). The filtrate of each section was tested for presence of phages and their titer was determined as previously described.

## Results

### Phage Isolation

In September 2014, plant and soil samples were collected for phage isolation from a field of watermelon showing symptoms of BFB. After enrichment of potential phages in bacterial host culture, 12 samples yielded phages able to lyse *A. citrulli* strains. Nine phage samples originated from the watermelon rhizosphere soil (*Acidovorax* phage ACF1 – 9), while three phage strains (*Acidovorax* phage ACF10 – 12) were isolated from watermelon leaves showing symptoms of BFB. After purification, the phage strains’ propagation resulted in titer ranging from 10^9^ to 10^10^ PFU/ml, respectively. All phages formed plaques 1.5–2 mm in diameter surrounded by 0.5 mm halo, after 24 h incubation with host *A. citrulli* KBI 86. Three phage strains, ACF1, ACF8, and ACF12 were selected for further characterization.

### Electron Microscopy

**FIGURE 1:**
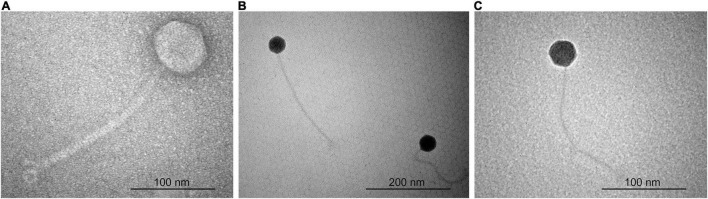
Transmission electron micrograph of *Acidovorax* phage ACF1 **(A)**, ACF8 **(B)**, and ACF12 **(C)**.

According to the particle morphology, i.e., presence of an icosahedral head and a long, flexible, non-contractile tail (Figure 1), observed by TEM, phages ACF1, ACF8, and ACF12 belong to the order *Caudovirales*, family *Siphoviridae*. Average head diameter of phage ACF1 was 64.4 nm, and length of tail was 220.9 nm. Phage ACF8 possesses head 45.8 nm in diameter and tail length 233.1 nm, while average dimensions of phage ACF12 were head 46.1 nm in diameter and tail 240.3 nm.

### Host Range Analysis

All tested phages were species specific. They lysed 30 *A. citrulli* strains tested, and showed no activity to two *A. citrulli* strains isolated in Serbia nor to the type strain NCPPB 3679*^T^*. However, the tested phage strains did not lyse any of the strains belonging to the different genera or less related bacterial species (Table 1).

### The Effect of Temperature, Chloroform, pH and Ultraviolet Irradiation on Phage Survival *in vitro*

*Acidovorax* phage ACF8 was inactivated at 66°C, while the point of thermal inactivation for phages ACF1 and ACF12 was 67°C. The chloroform treatment negatively affected vitality of the phage strains ACF1, ACF8, and ACF12, while the strain ACF1 was the most sensitive. After 1 h incubation with 20% chloroform, titer of phages ACF8 and ACF12 decreased by 11%, while ACF1 phage titer decreased by 36.38%.

**FIGURE 2:**
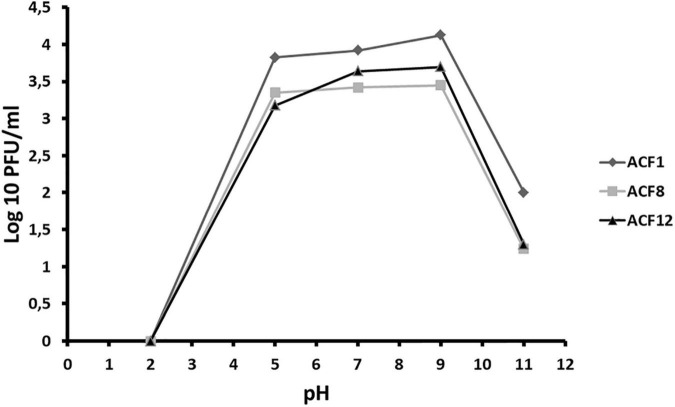
The effect of pH on *Acidovorax* phage ACF1, ACF8, and ACF12 vitality.

**FIGURE 3:**
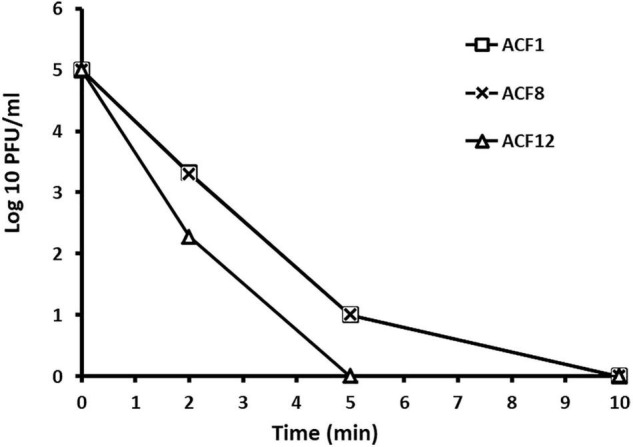
The effect of UV light on *Acidovorax* phage ACF1, ACF8, and ACF12 vitality.

Tested phages were stable at range of pH 5–9 (Figure 2), but were inactivated after 5 min (ACF12) or 10 min (ACF1 and ACF8) exposure to UV (Figure 3).

### Optimal Multiplicity of Infection, Adsorption Rate and One Step Growth Curve

**FIGURE 4:**
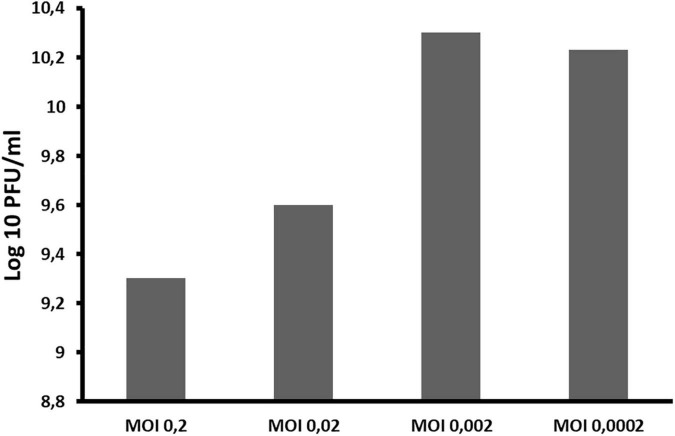
The influence of different ratios of bacteriophages and bacteria in suspension (MOI) during phage ACF1 multiplication.

The optimal MOI of ACF1 phage was determined to be 0.002 (Figure 4). The difference between the highest and the lowest phage titer produced by propagation at studied MOIs was 1 log unit.

**FIGURE 5:**
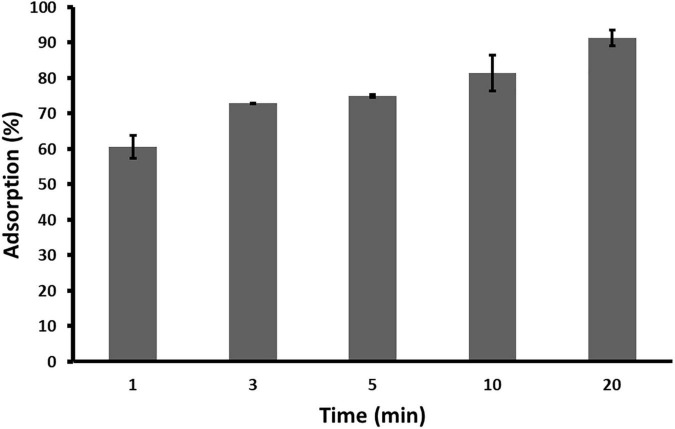
Adsorption rate of *Acidovorax* phage ACF1 to bacterial cell surface. Means and standard errors from three independent experiments are shown.

Adsorption rate of phage ACF1 to *A. citrulli* (KBI 86) cells after 20 min of incubation was 90% (Figure 5). Within 1 min, 60.65% of phages were adsorbed and the rate increased proportionally to the incubation time.

**FIGURE 6:**
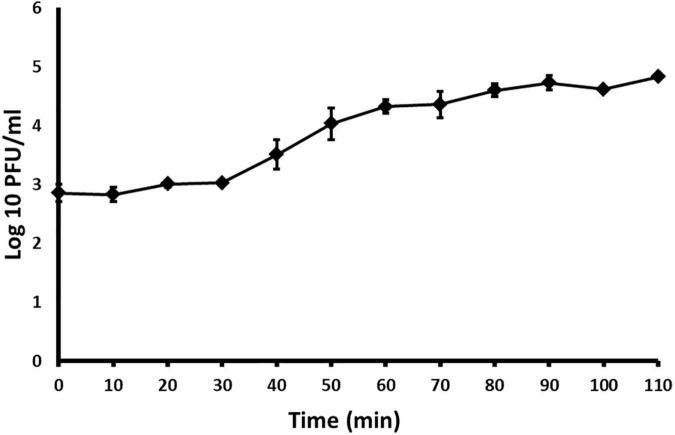
One-step growth curve of *Acidovorax* phage ACF1 propagated in *A. citrulli* KBI 86. Means and standard errors from three independent experiments are shown.

With the one–step growth test, the replication cycle growth curve was determined. Based on the results, the latent period of *Acidovorax* phage ACF1 is 30 min and burst size (average number of released phage virions per infected bacterial cell) is 74 ± 5 plaque forming units per infected cell (Figure 6). The rise period, when the number of phages increases due to release from the lysed cells, lasted ca. 60 min. The replication cycle of ACF1 in total lasted 90 min.

### Restriction Fragment Length Polymorphism Analysis of Phage DNA

Based on the RFLP analysis of phage’s DNA, it was determined that all three strains (ACF1, ACF8, and ACF12) possess identical restriction profiles after digestion with *Eco*RI and *Bsm*I enzymes. However, no DNA digestion occurred using the *Bam*HI enzyme, indicating that the *A. citrulli* phage genomes do not possess specific restriction sites for this enzyme (Supplementary Figure 1). RFLP profiles indicated genetic relatedness of all three phages.

### Phage ACF1 Genome Sequencing and Phylogenetic Analysis

Sequencing of *Acidovorax* phage ACF1 total DNA followed by genome assembly resulted in one long contig (∼60 kbp) and 152 short contigs (<1000 bp). Almost all the reads mapped to this long contig (99.9% of total bases) with high average coverage depth of 7194X. The average coverage depth of short contigs individually was relatively low (<20) and we therefore considered them as a contamination, which was also suggested by BLAST analysis. *In silico* RFLP analysis of the sequence corresponding to the large contig correlated to the patterns obtained by digesting phage DNA (data not shown). Overall, we considered that large contig represents nearly complete (draft) genome of the phage ACF1.

**FIGURE 7:**
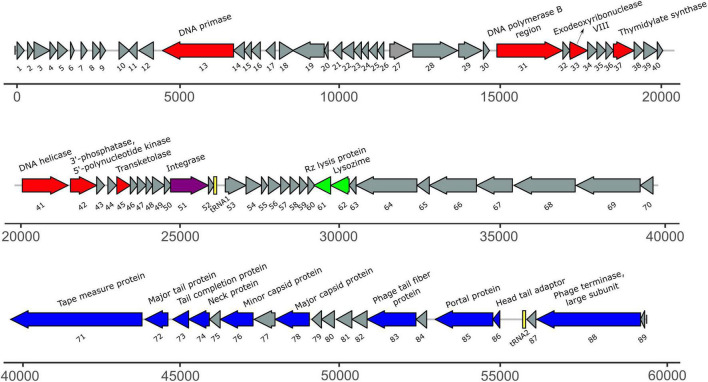
The annotated draft genome of *Acidovorax* phage ACF1. The ORFs coding for proteins involved in DNA replication/modification/transcriptional regulations are marked in red; ORFs coding for structural proteins are marked in blue; ORFs coding for enzymes involved in host lysis are marked in green; ORF coding for recombination protein is marked in purple. ORFs coding for hypothetical and phage proteins are marked in gray while tRNAs are marked in yellow. Arrows indicate the direction of transcription and translation. Unit of the presented draft genome annotation is in bp.

The size of the draft genome of phage ACF1 was 59.377 bp with a guanine-cytosine (GC) content 64.5%. The final annotation submitted to GenBank was based on combination of results obtained by RAST and DFAST, including additional functional annotation on basis of BLASTP, Pfam, Virfam, ARAGORN, and tRNAscan-SE searches. Taken together, a total of 89 open reading frames (ORFs) and two tRNAs (Ser- tRNA anticodon GCT and Ala-tRNA anticodon CGC) were identified (Figure 7). Length of ORFs ranges from 117 to 4077 bp, encoding putative proteins of 38–1358 amino acids. Total of 92.3% of the genome consists of coding regions. Among all identified ORFs, 74 start with ATG as the start codon while 14 start with GTG and one with TTG codon. The orientation of genome annotation showed that 45 genes are on the plus strand, while 44 on the reverse strand. Out of 89 putative ORFs, 20 (22.5%) have an assigned function, while the rest of 73 ORFs (77.5%) were classified as hypothetical or phage proteins (Supplementary Table 1 and Figure 7). Functional grouping of predicted ORFs revealed that nine ORFs encode phage structural proteins (tape measure protein, major tail protein, tail completion protein, neck protein, minor capsid protein, major capsid protein, tail fiber protein, portal protein, head-tail adaptor, and terminase large subunit), seven ORFs are involved in DNA replication/modification/transcriptional regulations (DNA primase, DNA polymerase B region, exodeoxyribonuclease VIII, thymidylate synthase, DNA helicase, 3′-phosphatase, 5′-polynucleotide kinase and transketolase), two ORFs coding the proteins involved in host lysis (Rz lysis protein, lysozyme), and one ORF coding for integrase protein was identified. The draft genome does not contain any genes encoding antibiotic resistance or toxins. Based on Virfam analysis of the sequences of head, neck and tail proteins, *Acidovorax* phage ACF1 was classified to belong to the family *Siphoviridae* of Type 1, Cluster 6 (Supplementary Figure 2), confirming previous observations by TEM.

**FIGURE 8:**
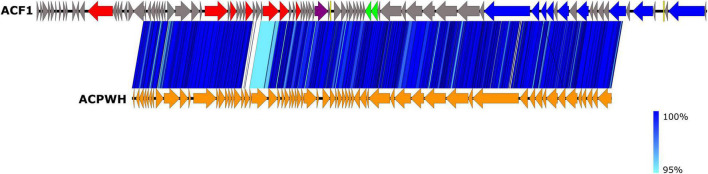
Comparison of draft genome sequence of *Acidovorax* phage ACF1 and *Acidovorax* phage ACPWH using Easyfig. Arrows indicate ORFs and their orientation. ORFs are colored according to their functions (see Figure 7). The blue vertical blocks between the genome maps indicate the level of identity between sequences (see the legend on the right) according to tBLASTx.

The BLASTn search using draft genome sequence of *Acidovorax* phage ACF1 showed the best match (99.23% identity and 71% query coverage) with the *Acidovorax* phage ACPWH (Gene Bank Acc no. MH727593.1). The relatedness between *Acidovorax* phages ACF1 and ACPWH was further confirmed by the high ANI value obtained between their genomic sequences (>99%). Most of the genes were highly conserved between these two phages (Figure 8). A total of 64 predicted proteins of *Acidovorax* phage ACF1 shared homology with ACPWH, while 25 proteins were unique to ACF1 phage, as evaluated by CoreGenes. Moreover, the *Acidovorax* phage ACF1 was compared to characterized genera from *Siphoviridae*, *Podoviridae*, and *Myoviridae* family using the PASC tool comparison, showing highest nucleotide identity of 15.85% to unclassified *Ralstonia* phage RS138 and 13.78% to *Xylella* virus Salvo (Sanovirus) both belonging to the *Siphoviridae* family. Moreover, ACF1 phage showed some nucleotide identity with Rauchvirus *Bordetella* virus BPP1 (13.57%) and *Xanthomonas citri* phage CP2 (13.52%) from *Podoviridae* family.

**FIGURE 9:**
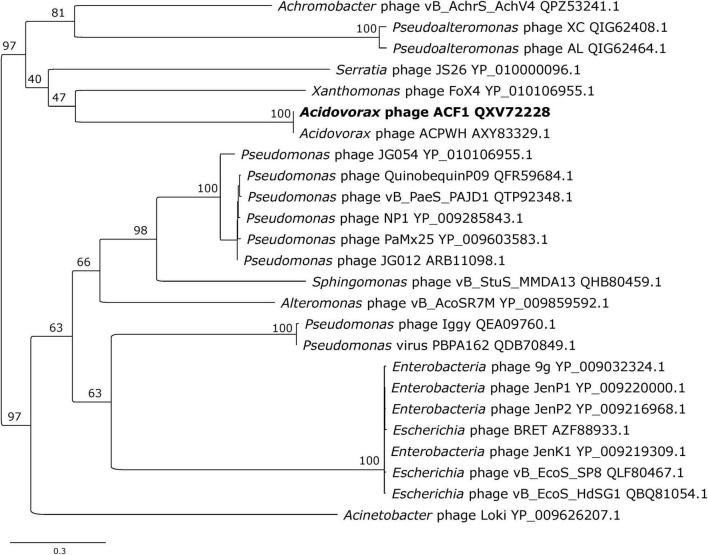
Maximum likelihood tree based on the DNA polymerase of *Acidovorax* phage ACF1 (bold font) and homologous proteins from other phages of the *Siphoviridae* family. The numbers on the nodes indicate ultrafast bootstrap values. The tree was midpoint rooted. The scale bar represents the number of expected substitutions per site under the best-fitting LG + I + G4 model.

Phylogenetic analysis of *Acidovorax* phage ACF1 based on DNA polymerase amino acid sequence revealed that ACF1 phage forms a clade with *Acidovorax* phage ACPWH that is distinct from the neighboring branch containing *Xanthomonas* phage FoX4 (Figure 9).

### Translocation of Phages Through Watermelon Plants

**FIGURE 10:**
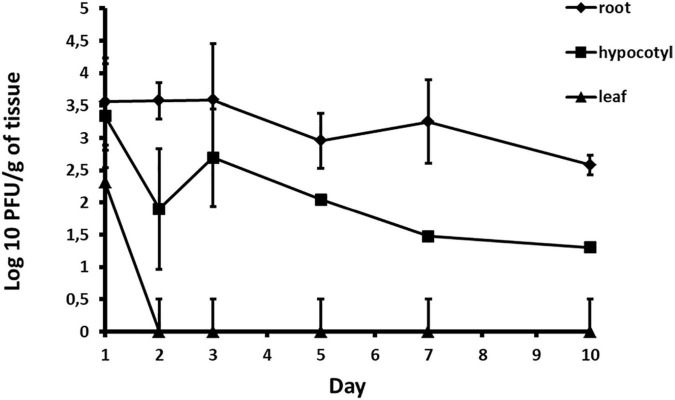
Quantification of phage translocation through plant tissue. The results shown are mean values of three replications with standard deviations.

*Acidovorax* phage ACF1 was detected in root, hypocotyl and leaf tissue 24 h after drenching the soil with phage suspension. The highest concentration of phages (6.03 × 10^4^ PFU/g) was detected in the root system 3 days after treatment (Figure 10). Phages remained both in hypocotyl and root tissue for 10 days, but were not detectable in leaf tissue after 48 h.

## Discussion

Due to the extensive use of pesticides and ineffectiveness of conventional and chemical control of bacterial diseases, there is an increased interest in biologically based solutions (Buttimer et al., 2017). Bacteriophages are gaining more attention and importance as potential biocontrol agents. Inadequate efficiency of available *A. citrulli* control methods requires innovative approaches. In this research, we studied several phage strains as potential biocontrol agents of *A. citrulli*. Particularly, we investigated their host range, survival in various conditions, replication cycle parameters and analyzed draft genome sequence of one representative phage. The ACF1 phage uptake by watermelon root system and translocation in upper parts was investigated as well.

All phages formed clear plaques 1.5–2 mm in diameter with 0.5 mm halo zone. As reported previously, the halo is often correlated with the presence of exopolysaccharide depolymerases, resulting in depolymerization of the EPS, which increases the lawn transparency (van Charante et al., 2021). Additionally, the halo zones might also be due to diffusion of virus-encoded non-virion associated lytic enzymes such as endolysins, which degrade the cell wall of neighboring cells (Jurczak-Kurek et al., 2016).

As much as specificity of phages allows safe application as antimicrobials, without affecting other bacteria present in the environment, high specificity could be also one of the disadvantages for their application in biocontrol of plant pathogenic bacteria since they might be limited to infecting only particular strains of the target bacterial species (Jones et al., 2007). One way of expanding the phage treatment host range is using cocktails of different phage strains covering broader range of the host bacterium strains. The cocktail may contain phages targeting different receptor sites on the bacterial cell surface or affecting the biofilm formation. Phage cocktails also could reduce a chance of resistance development in pathogenic bacteria, considering low possibility of developing resistance simultaneously to multiple phages attacking the target bacterium (Choudhary et al., 2022). The fact that two *A. citrulli* strains were resistant to all 12 phage strains indicated a weak point in the phages’ host range. Prior attempts for controlling the disease in a field, variation of the phage host range and possibility of expanding it must be studied in detail to avoid favoring of the resistant bacterial strains.

Results of one-step growth and adsorption studies, including draft genome analysis, support the use of *Acidovorax* phage ACF1 as biocontrol agent. ACF1 phage has a burst size of 74 ± 5 phage particles per infected cell and rapidly adsorbs to the host cells, with 90% of phages being attached after 20 min. Sensitivity of ACF1, ACF8, and ACF12 to chloroform and UV light could be an additional disadvantage that requires a solution, in regard of their application. Reduced viability of phages of *Dickeya solani, Erwinia amylovora*, and *Xanthomonas euvesicatoria* after treatment with UV light or chloroform has been already reported (Gašić et al., 2011; Czajkowski et al., 2014; Born et al., 2015). Chloroform is used in the process of phage isolation or multiplication in order to eliminate unlysed and phage resistant bacterial cells, especially if the volume of the phage suspension does not justify application of the filtering procedure (Clokie and Kropinski, 2009). Cost-effectiveness and feasibility of production and storage of phage preparations are some of the conditions that should be met. Sensitivity to UV illumination and low pH of ACF1, ACF8, and ACF12 is a shared trait with other bacteriophages of plant-associated bacteria. Jones et al. (2007) demonstrated that factors such as exposure to sunlight, high temperatures, extreme pHs and high ionic concentration inactivate phages and impose a practical problem for their application in agriculture.

Phage characterization by TEM revealed that isolated phages belong to the order *Caudovirales*, and the family *Siphoviridae* based on their head and tail morphology. Among all identified viruses, the *Caudovirales* or tailed phages are the most numerous. Ackermann and Prangishvili (2012) reported that 96.3% of the described phages belonged to the tailed phages, while only 230 (3.7%) are polyhedral, filamentous, or pleomorphic. Moreover, the family *Siphoviridae*, is the largest family with over 3600 descriptions, or 57.3% (Ackermann and Prangishvili, 2012). Size of studied phages corresponds to the size indicated for *Siphoviridae* (head 40–80 nm, tail 5–10 × 100–210 nm and genome size 21–134 kb) in the ICTV 9th report (King et al., 2011). So far, there are two described phages of *A. citrulli*, ACP17 belonging to the *Myoviridae* family (Rahimi-Midani et al., 2018), and phage ACPWH which belongs to the *Siphoviridae* family (Rahimi-Midani et al., 2020). Size of the phage ACF1 was similar to the phage ACPWH (head is 55 ± 5 nm in width and 60 ± 5 nm in length, with a tail length of approximately 180 ± 5 nm) while the other two studied strains had smaller head and longer tail compared to the phage ACPWH.

Draft genome of *Acidovorax* phage ACF1 obtained in this study has a size of 59.377 bp, with 89 ORFs and two tRNA genes (Figure 7 and Supplementary Table 1). ACF1 phage displayed a very high genomic identity with previously described *Acidovorax* phage ACPWH isolated in South Korea (Rahimi-Midani et al., 2018), as indicated by BLASTn and ANIb analyses. Nevertheless, the *Acidovorax* phage ACPWH, specific to *A. citrulli*, has a dsDNA genome of 42.499 bp which is smaller than the genome of ACF1, and encodes 64 ORFs, with no tRNA gene. The draft genome annotation showed that ACF1 has all basic structural and functional genes coding DNA replication proteins, host lysis proteins, and tail structure proteins. Moreover, gene with homology to known integrases was detected in the draft genome of ACF1. The presence of integrase in the phage genome suggests that the phages are able to integrate their genomes into the host genome and enter the lysogenic phase (Groth and Calos, 2004). Phage possessing lysogenic life cycle (temperate phages) are not considered suitable for therapeutic purposes due to their potential to transfer bacterial antibiotic resistance or virulence genes via transduction (Monteiro et al., 2019). However, recent advances in synthetic biology could enable engineering of temperate phages toward elimination of genes involved in a lysogenic life cycle or in bacterial virulence (Zhang et al., 2013; Pires et al., 2016; Kilcher et al., 2018). In this way temperate phage could become obligatory lytic and suitable for use in phage therapy (Monteiro et al., 2019). Lytic phages typically contain a DNA polymerase gene and other genes associated with DNA replication such as DNA primase and DNA helicase (Lohr et al., 2005). However, most of the lysogenic phage genomes of members of the *Siphoviridae* and *Myoviridae* do not possess these DNA replication genes (Lohr et al., 2005). The presence of an integrase gene, but also, DNA replication genes in the draft genome of phage ACF1, suggested that phage ACF1 might have some pseudolysogenic characteristics (Abedon, 2009; Cenens et al., 2013).

CoreGenes analysis showed that 64 protein-coding sequences shared homology with ACPWH phage, while 25 were unique to *Acidovorax* phage ACPWH. This is in accordance with the difference in size of two phages, since phage ACPWH possess 25 ORFs less then phage ACF1. Additionally, PASC analysis showed that ACF1 has common genes (13–15%) with *Ralstonia* phage RS138 and *Xylella* virus Salvo from *Siphoviridae* family, and *Bordetella* virus BPP1 and *Xanthomonas citri* phage CP2 (13.5%) from *Podoviridae*.

Since there is no universal gene present in all phages, some of the signature gene products could be used for study of virus diversity (Adriaenssens and Cowan, 2014). Phylogenetic analysis of *Acidovorax* phage ACF1 based on DNA polymerase amino acid sequence grouped together with phage ACPWH, near *Xanthomonas* phage XoF4.

Automated classification of tailed bacteriophages according to their neck organization by Virfam (Lopes et al., 2014), revealed that *Acidovorax* phage ACF1 belongs to *Siphoviridae* of Type 1, Cluster 6 (Supplementary Figure 2). Neck Type 1 phage genomes contain the following proteins: portal protein, Adaptor of type 1 (Ad1), Head-closure of type 1 (Hc1), Neck protein of type 1 (Ne1), and tail-completion of type 1 (Tc1). Cluster 6 is mostly composed of siphophages with small genome sizes that infect Proteobacteria and possess a different head-neck-tail gene order compared to that observed in the majority of siphoviruses. As it seen in Figure 7, gene coding for head-tail adaptor (Ad1) is positioned between the genes coding for the terminase and portal proteins, while in most Type 1 siphoviruses, Ad1 is usually located downstream from the major capsid protein gene.

Instead of treating the phyllosphere parts and expose phages to UV light, in this study we tested possibility of applying phage suspension by drenching the soil. It was determined that phage ACF1 can be absorbed from soil by the root system of watermelon plants and transported up to the leaves. The titer of phages was most stable in the root system and did not drop until 4 days after the phage treatment. Phages detected in the leaves were of a lower titer and endured shorter than in other parts of the plant. The highest titer of phages detected in the three trials was 6.03 × 10^4^ PFU/g, which is 4.37 log units lower than the applied phage concentration (1.4 × 10^9^ PFU/ml). This information and the variation of the detected titer values indicate the presence of obstacles in the absorption of phages. Previous research has shown that phages could be immobilized by biofilm formed in the substrate (Storey and Ashbolt, 2001), adsorbed to soil particles (Williams et al., 1987), as well as inactivated by low pH values of the substrate (Sykes et al., 1981). Also, the concentration of the phage suspension relative to the volume of rhizosphere to which it is applied significantly affects the detected concentration of phage, their persistency in plant tissue and efficacy of translocation. Iriarte et al. (2012) have shown that *Ralstonia solanacearum*, *Xanthomonas perforans*, and *X. euvesicatoria* phages were transported through the vascular elements of tomato plants up to the leaves. Furthermore, they showed that the translocation of phages can be affected by the type of phage, plant species, plant age, plant size and the type of substrate in which the plant was grown. Rahimi-Midani and Choi (2020) recently studied the transportation of *A. citrulli* phages throughout melon plants. They tracked the transport of phages through melon plant parts by PCR and fluorescent microscopy (Rahimi-Midani et al., 2020). The study demonstrated that phages applied to the soil can translocate through the melon plant vascular tissue up to leaves and decrease the disease severity to 27% and increase survival of the infected plants to 100%.

Our results indicated that considering lytic life cycle and some of the biological characteristics of investigated phage strains, they possess potential in control of watermelon fruit blotch pathogen. However, before going to the field, the risks such as host range, survival in an open environment, type and frequency of application, need to be addressed properly. Otherwise the application of these biocontrol agents may be compromised.

## Data Availability Statement

Data available at ncbi.nlm.nih.gov/nuccore/2071735446 BioProject PRJNA745195.

## Author Contributions

KG and AO conceived and designed the experiments. KG, MO, NK, NZ, MI, and DR performed the experiments. KG, NK, and MO analyzed the data. KG and MO wrote the manuscript. AO revised the manuscript. All authors read and approved the final manuscript.

## Conflict of Interest

The authors declare that the research was conducted in the absence of any commercial or financial relationships that could be construed as a potential conflict of interest.

## Publisher’s Note

All claims expressed in this article are solely those of the authors and do not necessarily represent those of their affiliated organizations, or those of the publisher, the editors and the reviewers. Any product that may be evaluated in this article, or claim that may be made by its manufacturer, is not guaranteed or endorsed by the publisher.

## Footnotes

1
http://www.acaclone.com

2
https://dfast.ddbj.nig.ac.jp/

3
https://blast.ncbi.nlm.nih.gov/Blast.cgi?PAGE=Proteins

4
http://pfam.xfam.org

5
http://biodev.cea.fr/virfam/

6
https://cge.cbs.dtu.dk/services/VirulenceFinder/

7
https://cge.cbs.dtu.dk/services/ResFinder/

8
http://130.235.46.10/ARAGORN/

9
http://lowelab.ucsc.edu/tRNAscan-SE

## Supplementary Material

Supplementary Figure 1 Restriction DNA profiles of *Acidovorax* phage ACF1, ACF8, and ACF12 after digestion with enzymes *Eco*RI and *Bam*HI, M1 – Lambda DNA/*Eco*RI + *Hin*DIII Marker (Thermo Fisher, Lithuania), M2 – GeneRuler DNA Ladder Mix (Thermo Fisher, Lithuania).

Supplementary Figure 2 Automated classification of *Acidovorax* phage ACF1 according to its neck organization by Virfam analysis (Lopes et al., 2014). The different branches of the tree were sorted into 10 Clusters, highlighted by different background colors and numbers.

Supplementary Table 1 Putative open reading frames (ORFs) of *Acidovorax* phage ACF1, their predicted functions and detected conserved domains.
