# Supplementary figures and images for: Isolation, Characterization and Draft Genome Analysis of Bacteriophages Infecting Acidovorax citrulli
Source: Front Microbiol. 2022 Feb 3;12:803789. doi: 10.3389/fmicb.2021.803789 (PMC8851203; doi:10.3389/fmicb.2021.803789)

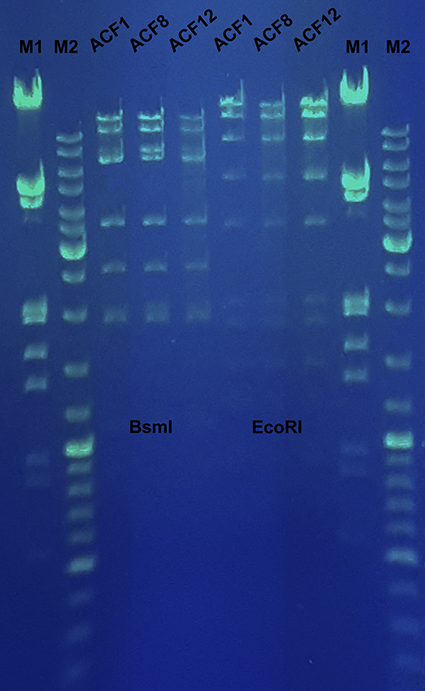

Supplement: Supplementary Figure 1 — Restriction DNA profiles of Acidovorax phage ACF1, ACF8, and ACF12 after digestion with enzymes EcoRI and BamHI, M1 – Lambda DNA/EcoRI + HinDIII Marker (Thermo Fisher, Lithuania), M2 – GeneRuler DNA Ladder Mix (Thermo Fisher, Lithuania). [file Image_1.TIF]

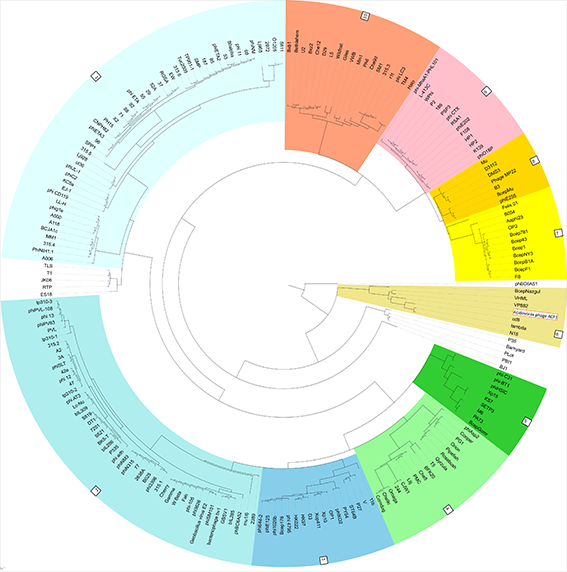

Supplement: Supplementary Figure 2 — Automated classification of Acidovorax phage ACF1 according to its neck organization by Virfam analysis (Lopes et al., 2014). The different branches of the tree were sorted into 10 Clusters, highlighted by different background colors and numbers. [file Image_2.TIFF]
